# The National Pension Fund for Nurses and Hospital Officials

**Published:** 1888-01-21

**Authors:** 


### THE NATIONAL PENSION FUND FOR NURSES AND HOSPITAL OFFICIALS.

THE following leading article appeared in the *Times* on Friday, 13th inst., and it will no doubt be read with much pleasure by all our readers :—

WE announce this morning with great satisfaction that the sum of £20,000, which is required for the foundation of a Pension Fund for Nurses and Hospital Officials, has been contributed by the munificence of four London merchants, Messrs. Gibbs, Hambro, J. S. Morgan, and Rothschild. The necessity of establishing such a National Fund has long been recognized by persons interested in hospital economy, and, thanks mainly to the persistent and disinterested efforts of Mr. Henry C. Burdett, the scheme has already been put in a practical working shape. A meeting of the Hospitals Association was held on October 12th last year, in the rooms of the Society of Arts, at which Mr. Burdett propounded and explained his scheme. Sir Andrew Clark occupied the chair, and warmly commended Mr. Burdett's proposals. After a prolonged discussion resolutions were unanimously passed approving the scheme, and referring it to the Council of the Association, in order that they might take such steps as might be necessary to establish the proposed fund with as little delay as possible. It was necessary, however, in order to comply with the requirements of the law, that £20,000 should be deposited with the Court of Chancery prior to incorporation, as a preliminary to the establishment of the fund on an adequate basis. For some time hopes were entertained that this sum might be obtained, with her Majesty's consent, from the Women's Jubilee Offering. These hopes were, however, disappointed by the report of the committee nominated to consider and advise upon the best application of the Jubilee Offering to nursing purposes in accordance with her Majesty's wishes, and it now turns out that the momentary disappointment was a blessing in disguise. The Jubilee Offering remains unimpaired in amount to be applied to the wise and beneficent purposes recommended by the Committee, and the National Pension Fund for Nurses and Hospital Officials is none the poorer. No sooner was the report of the Committee on the Women's Offering made public than Mr. Burdett, nothing daunted, put himself in communication with some of the leading merchants of the City, and explained to them the purposes and needs of the National Pension Fund. In a very few days he received the munificent offer to provide the required sum of £20,000 from the four merchants above-mentioned.

We need not insist on the great advantage of establishing such a fund as is proposed by Mr. Burdett. The scheme is approved by The Hospitals Association, and the splendid gift of the four merchants furnishes twenty thousand excellent and convincing arguments in its favour. "For all practical purposes," said Mr. Burdett in his paper read at the meeting of October 12th, "no attempt has yet been made to provide adequately for the sustenance and care of nurses, much less of hospital officials, when permanently invalided or when too old for duty, such provision as exists being very limited in quantity, and not readily available, because, where existent, removal to a new sphere of duty disqualifies the members." What is needed, then, is a fund self-supporting in principle, and therefore financially impregnable, and sufficiently comprehensive to include and provide for all nurses and hospital officials who choose to avail themselves of it. When once established on a permanent and sound basis the scheme should need only the confidence and co-operation of those for whose benefit it is established to make it self-acting and self-supporting. Each person who joined the fund would be enabled by payment of the annual premium specified in tables prepared for the purpose to secure either sick allowance, or the amount of pension which each desired to obtain on attaining a certain age. It might thus seem at first sight as if the fund would be self-supporting from the outset and no external contributions would be necessary. This, however, is not so. The law requires that £20,000 should be deposited with the Court of Chancery to begin with. The financial basis of the scheme cannot be secured without this preliminary. Besides, every external contribution to the fund will *pro tanto* place the beneficial contributors in a more advantageous position. In principle the fund should be self-supporting: that is, the sum obtained by the beneficiary should be the actuarial equivalent, neither more nor less, of the premiums paid. The £20,000 required as a foundation must be invested in the hands of trustees, and only its interest can go to the strengthening of the Fund, so as to give the beneficiaries as large an equivalent for their premiums as is consistent with impregnable financial stability. There are, however, many ways in which external contributions may be made available without impairing the self-supporting character of the Fund. Hospital authorities, for instance, might undertake to pay at their discretion a portion of the premiums for members of the staff they employ. Benevolent persons in general might contribute to the Fund, so as to provide bonuses over and above the annuities secured by the premiums to all the beneficiaries equally. Or again, patients and others who desired to requite the services rendered to them in sickness by a particular nurse might give a donation to the Fund, to be applied for the benefit of the nurse in question, by way of either reducing her premium or increasing the amount of her pension. In all these ways public benevolence, whether general or individual, might be turned to the advantage of the scheme without derogation to its essential feature, namely, its provident and self-supporting character. As the foundation is now secured by the munificence which we record to-day, it only remains to set the scheme on foot in practical and working order. This depends mainly on the co-operation of those for whose benefit the Fund is to be established. There are, it is calculated, some 25,000 nurses and hospital officials now employed in this country who would be eligible as beneficiaries of the Fund. Of these some 1,500 have already registered their names as anxious to join the Fund, and now that the Fund is actually started we should anticipate that this number would rapidly increase. Provision has been made, however, for the return of the £20,000 to the donors and the winding up of the Fund if proposals for granting at least 1,000 annuity policies have not been received by the 1st of January, 1890. This provision is reasonable and judicious, but it is hardly likely to take actual effect. It would be a poor return for the prompt munificence of the four merchants and the untiring zeal of Mr. Burdett if a Fund so wisely conceived and so liberally supported should fail for want of appreciation and co-operation on the part of those for whose benefit it is established.

---

THE Queen's Committee appointed to consider the method by which the surplus of the Women's Jubilee Offering may best be applied for the promotion of Nursing, have reported in favour of the foundation of an institution for promoting the education and maintenance of nurses for the sick poor in their own homes. Her Majesty having approved this report, which was made public on the 7th inst., it became necessary to consider what could be done to help the great army of nurses, numbering some twenty-five thousand, who have to nurse the sick poor in public institutions, and the families of the upper and middle classes in their own homes.

These devoted women ought to be provided for in their old age or when attacked by disease, but no National Pension and Sick Fund can be established by law upon an adequate basis until £20,000 has been deposited in the Court of Chancery, prior to the incorporation of any such society. THE HOSPITAL is therefore happy to be authorized to state that when it became known that this money would not be forthcoming from the Queen's Fund, Mr. Burdett communicated the National Pension scheme to some of the leading merchants in the City, four of whom, Messrs. Gibbs, Hambro, J. S. Morgan, and Rothschild, have generously agreed to provide the necessary £20,000.

Steps have been taken to incorporate the National Pension Fund, with the object, amongst others, of granting to nurses and other hospital officials annuities, as well as allowances during sickness, so as to secure an adequate provision to those whose lives are devoted to the care of the sick when their period of work is finished, or when they themselves are attacked by illness. Except in respect to a few minor details, all the arrangements for the establishment and incorporation of the Fund are now completed, and letters, addressed to the Acting Secretary, the National Pension Fund, 38, Old Jewry, E.C., will receive prompt attention. Although 1,500 nurses and hospital officials have registered their names as anxious to join the Fund, it has been thought desirable to provide in the deed of incorporation that unless proposals for granting one thousand annuity policies are received and accepted before the expiration of two years from January 1st, 1888, the £20,000 will be returned to those who have so generously provided it, and the Fund will be wound up. This proviso will enable the managers to ascertain to what extent the nurses and officials desire to be provided for, and all who do will not delay in sending in their applications.

Provision has also been made for the establishment of a Bonus Fund, to which contributions may be made by persons interested in nurses and by the institutions who engage and pay them, and already a considerable sum has been promised. Contributors to the Bonus Fund will constitute the members of the society and will elect half the council, the other half being elected by the nurses and others who may become annuitants of the Fund.

Copies of the constitution, prospectus, forms of application, and other necessary papers will be ready by the end of the month, when we propose to publish the whole scheme in full.

### ANOTHER MUNIFICENT GIFT TO THE FUND.

THE noble liberality of the four City princes who subscribed the £20,000 for the starting of the Pension Fund has had a most wholesome and stimulating effect. Offers of help are now coming in freely from grateful patients and others. Among the rest, Mr. Edward F. Coates, of Ewell, Surrey, writes :

"As one of those deeply interested in the success of the National Pension Fund for Nurses and Hospital Officials, I am indeed glad to hear that there is every probability that the £20,000 required to be deposited with the Court of Chancery by the Life Assurance Act of 1870, will be forthcoming. If such is the case, I hope you will permit me to take my share in the good work by contributing a sum of two hundred and fifty guineas to the Bonus Fund to provide for the preliminary and all working expenses up to the end of the first year. I have fixed the sum at two hundred and fifty guineas, because I understand that the actuary states that that amount will prove more than enough to provide all the expenditure which is necessary to give the movement an adequate commencement, and I can only hope that the Fund will prove in every way a boon and a blessing to the large and devoted army of workers who spend their lives in ministering to the sick and helpless in this country."

### GRATEFUL NURSES.

E. P. WRITES : "We, nurses, feel we should like to thank the four gentlemen who have been so generous to our community with regard to the National Pension Fund, but not knowing their addresses, we are compelled to trouble you to address them for us, and hope you will not think it too much trouble. I have stamped the envelopes, and left one open for you to read. Such kindness merits our humble acknowledgment. With thanks to you also, Sir, we are your obedient servants, NURSES."

